# Application of omics technologies in studies on antitumor effects of Traditional Chinese Medicine

**DOI:** 10.1186/s13020-024-00995-x

**Published:** 2024-09-09

**Authors:** Peng Tan, Xuejiao Wei, Huiming Huang, Fei Wang, Zhuguo Wang, Jinxin Xie, Longyan Wang, Dongxiao Liu, Zhongdong Hu

**Affiliations:** 1 School of Chinese Materia Medica, Beijing University of Chinese Medicine, Beijing, 100029 China; 2 Modern Research Center for Traditional Chinese Medicine, Beijing Research Institute of Chinese Medicine, Beijing University of Chinese Medicine, Beijing, 100029 China

**Keywords:** Traditional Chinese Medicine, Antitumor, Omics technologies, Mechanism investigation

## Abstract

Traditional Chinese medicine (TCM) is considered to be one of the most comprehensive and influential form of traditional medicine. It plays an important role in clinical treatment and adjuvant therapy for cancer. However, the complex composition of TCM presents challenges to the comprehensive and systematic understanding of its antitumor mechanisms, which hinders further development of TCM with antitumor effects. Omics technologies can immensely help in elucidating the mechanism of action of drugs. They utilize high-throughput sequencing and detection techniques to provide deeper insights into biological systems, revealing the intricate mechanisms through which TCM combats tumors. Multi-omics approaches can be used to elucidate the interrelationships among different omics layers by integrating data from various omics disciplines. By analyzing a large amount of data, these approaches further unravel the complex network of mechanisms underlying the antitumor effects of TCM and explain the mutual regulations across different molecular levels. In this study, we presented a comprehensive overview of the recent progress in single-omics and multi-omics research focused on elucidating the mechanisms underlying the antitumor effects of TCM. We discussed the significance of omics technologies in advancing research on the antitumor properties of TCM and also provided novel research perspectives and methodologies for further advancing this research field.

## Introduction

Tumors are characterized by abnormal proliferation of normal cells in the body, caused by a combination of various internal and external factors; malignant tumors are commonly known as cancers [1]. According to global cancer statistics, in 2020, approximately 19.3 million cases of cancer were newly diagnosed globally, and nearly 10 million deaths were recorded [2]. The current existing methods to combat tumors primarily involve the use of radiotherapy, chemotherapy, or surgical resection. Although radiation therapy is effective in treating various solid tumors including breast cancer, lung cancer, and colorectal cancer, it can also cause harm to normal tissues, leading to various adverse reactions (e.g., radiation pneumonitis, enteritis, lymphopenia) and increasing the risk of tumor recurrence and metastasis [3, 4]. The long-term repeated administration of chemotherapeutic agents leads to the development of acquired resistance (e.g., overexpression of P-glycoprotein and multidrug resistance-associated protein genes) and significant toxicity, thereby greatly limiting their clinical utility [5]. The above reasons often lead to treatment failure or poor prognosis for cancer patients.

**Fig. 1:**
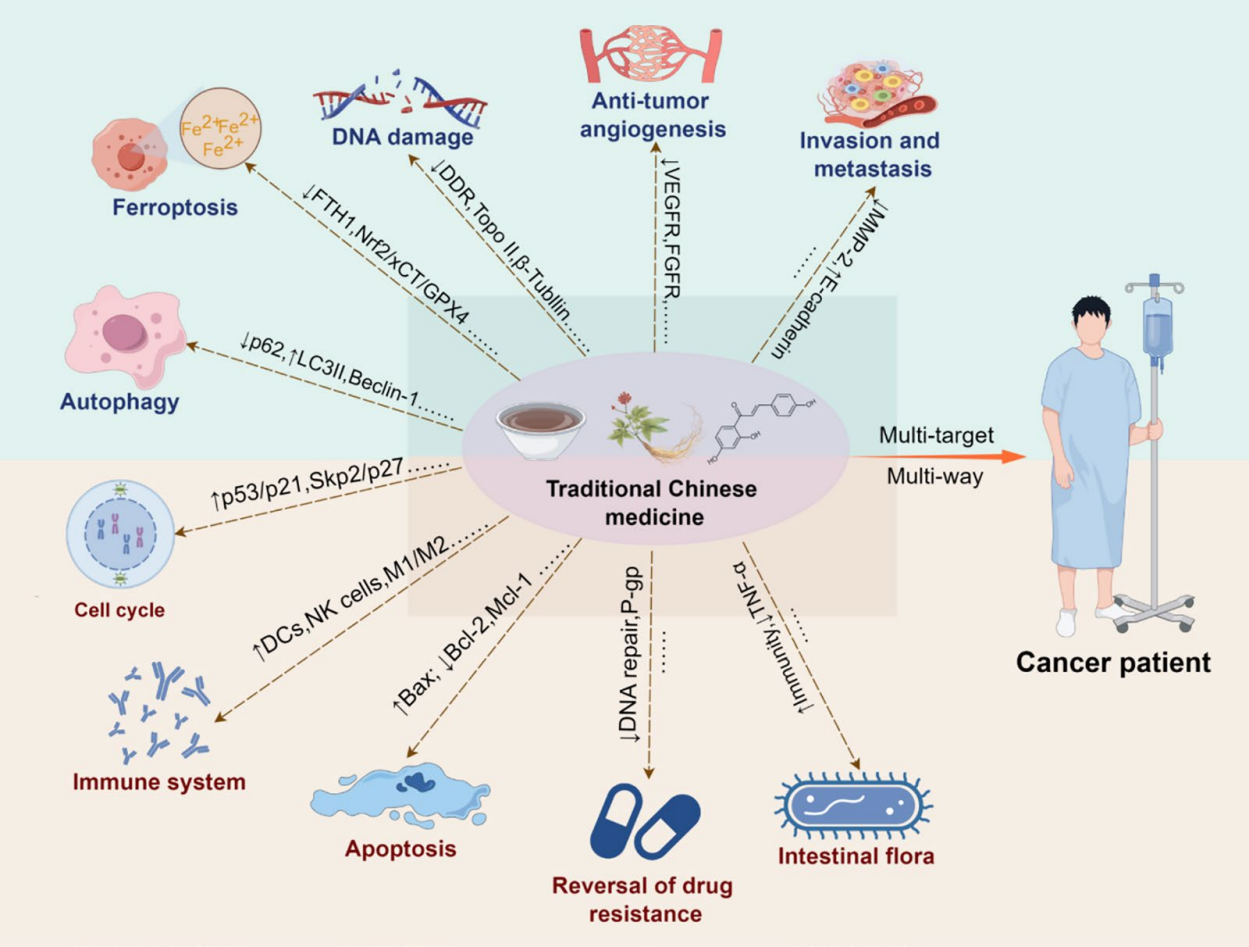
Traditional Chinese Medicine (TCM) exerts anti-tumor effects through multiple pathways and targets. TCM has the characteristics of "multi-component, multi-target, multi-pathway". It plays an anti-tumor role by inhibiting tumor cell proliferation, promoting tumor cell apoptosis, improving immune response, reversing drug resistance and other ways

Traditional Chinese medicine (TCM) is an indispensable part of public health in China and is gradually gaining acceptance in Western countries [6]. In TCM theory, tumors are considered to be “pathogenic factors” or “evil.” Research has indicated that numerous traditional Chinese herbs, compounds, and their active components exhibit antitumor activities. For example, the main component of *Astragalus membranaceus*, i.e., astragalus polysaccharide, can exert antitumor effects through multiple pathways, such as cell cycle regulation, enhancement of immune function, and reversal of drug resistance [7, 8]. Xihuang pill is a well-known classic anti-cancer formula used in clinical practice. It shows favorable effects on various malignant tumors and is commonly used as an adjuvant therapy to improve patient prognosis [9–11]. Traditional Chinese herbs and formulations exhibit intricate chemical compositions. This complexity often leads to synergistic effects of multiple active ingredients interacting with multiple molecular targets ultimately exerting antitumor effects through multiple pathways (as shown in Fig. 1). Therefore, traditional studies that focus only on a single target or pathway cannot comprehensively elucidate the complex antitumor mechanisms of TCM [12]. This significantly hinders the further development of anti-tumor Chinese herbal medicine and fails to benefit clinical patients.

Omics technologies refer to an integrated approach that combines multiple high-throughput technologies and data analysis methods to measure and analyze various molecular levels within an organism, revealing interactions and functional associations among different molecules, including genomics, proteomics, transcriptomics, and other related techniques [13]. With the continuous advancement in high-throughput sequencing, liquid chromatography-mass spectrometry (LC–MS), gas chromatography-mass spectrometry (GC–MS), and other technologies, omics studies are generating extremely rich datasets, which provide new methods for investigating the mechanism of action of TCM and contribute to the modernization of TCM [14]. Currently, a significant amount of research on TCM for anticancer purposes has greatly benefited from omics technologies, particularly transcriptomics and proteomics. By comparing changes in overall transcript levels or protein levels in tumor cells or tissue samples and conducting enrichment analysis, researchers can identify potential pathways or targets through which TCM exerts its anticancer effects, thereby providing guidance for future studies. Additionally, muti-omics technologies can be utilized at various levels to elucidate the mechanisms of action of TCM in anticancer treatment; for instance, the combination of metagenomics and metabolomics can effectively illustrate how TCM indirectly exerts its anticancer effects through the gut microbiota. These approaches align with the holistic concept of TCM, which emphasizes a comprehensive understanding and treatment of diseases. The omics technologies will facilitate the elucidation of the underlying mechanisms behind the significant anti-tumor effects of TCM, thereby advancing its further clinical applications, particularly for Chinese medicine formulas.

In this review, we provided a comprehensive overview of the applications of omics technologies in antitumor research in the field of TCM, including transcriptomics, proteomics, metabolomics, microbiomics, single-cell omics, and integrated multi-omics approaches. We emphasize their significance and limitations in this field to enhance their utilization.

## Omics technologies

**Fig. 2:**
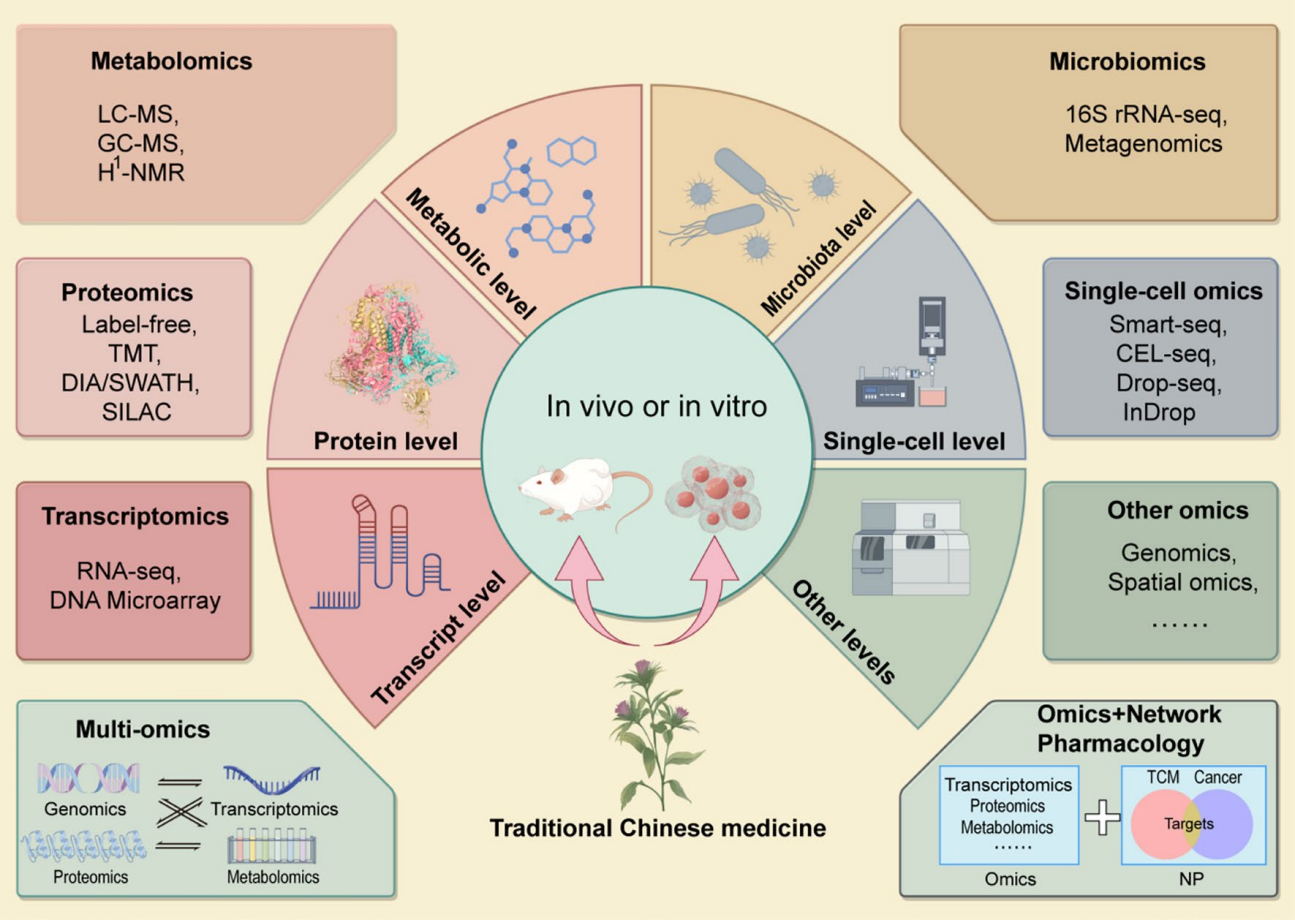
The application of omics technologies in anti-tumor research of traditional Chinese Medicine (TCM).The intervention of TCM in cancers leads to changes in mRNA, protein and other biological levels. These changes can be detected by RNA-seq, SILAC, 16S rRNA-seq and other histological techniques and help to reveal the complex anti-tumor network of TCM

In 1958, Crick proposed the “Central Dogma” after discovering the double-helical structure of deoxyribonucleic acid (DNA). He detailed the process of genetic information transcription from DNA to RNA and its translation into proteins in living organisms [15]. Genomics, transcriptomics, proteomics, and metabolomics represent the four levels of genetic information corresponding to replication, transcription, translation, and regulation, respectively, in the Central Dogma. However, traditional omics studies are based on mixed cell populations, which makes it challenging to obtain differential information and spatial positioning information of different types of cells [16]. Single-cell omics technologies were innovated as a solution to address this issue. These advancements enabled thorough analysis on a large scale across various dimensions such as genomics, transcriptomics, proteomics, and other levels in each cell in the sample [17]. Omics technologies can be used to accurately predict tumor biomarkers [18], novel therapeutic targets [19], and tumor heterogeneity [20]. They can also be used to elucidate the mechanisms of antitumor drugs [21]. The commonly applied omics technologies in current studies on antitumor mechanisms of TCM include transcriptomics, proteomics, metabolomics, microbiomics, and single-cell omics. The main techniques and applications of these techniques are summarized in Fig. 2.

### Transcriptomics

Transcriptomics research methodologies encompass DNA microarray, employing hybridization techniques, and various high-throughput sequencing techniques. Commonly used DNA microarray platforms are further classified into complementary deoxyribonucleic acid (cDNA) microarrays and oligonucleotide microarrays, based on the probe molecules used [22]. The advent of next generation sequencing (NGS) has significantly advanced transcriptomics in the post-genomic era by overcoming challenges related to throughput and cost, which were prevalent prior to the technological advancements in sequencing methodologies [23]. RNA sequencing (RNA-seq), in which the entire cDNA of a sample is sequenced using NGS, is widely performed in the field of transcriptomic research following TCM intervention. Compared to other methods, such as those involving microarrays, RNA-seq offers high sensitivity and accuracy [24]. The commonly used representative NGC platforms include Roche 454, Illumina, BGI Genomics, etc. [25, 26]. To address the limitations of short read lengths, template switching during polymerase chain reaction (PCR) amplification, and difficulties in analyzing complex repetitive sequences associated with NGS, third-generation sequencing technologies, such as single-molecule real-time sequencing (SMRT-seq) and nanopore single-molecule sequencing, have emerged as promising alternatives [27].

### Proteomics

Due to the significant advancements in protein/peptide enrichment techniques, isotope labeling methods, and mass spectrometry technology, substantial progress has been made in qualitative and quantitative proteomics based on mass spectrometry [28]. Quantitative proteomics is the core of proteomic techniques and one of the most common approaches used to study the antitumor effects of TCM. Commonly used quantitative techniques include label-free quantification proteomics [29], data independent acquisition/sequential window acquisition of all theoretical spectra (DIA/SWATH) proteomics [30, 31] and isotope labeling-based tandem mass tags/isobaric tag for relative absolute quantitation (TMT/iTRAQ) and stable-isotope labelling by amino acids in cell culture (SILAC) proteomics [32–34]. The major difference between the two proteomic strategies lies in whether isotopic labeling is applied to proteins or peptides. In label-free quantification techniques, proteins are directly extracted, enzymatically digested, and then, analyzed using LC–MS. Relative quantification is performed based on the peak area or capture frequency of peptides. This method is cheap and avoids potential errors introduced by labeling and is suitable for analyzing differential protein expression in large-scale samples [29]. Unlike label-free quantification, TMT/iTRAQ involves isotopic labeling of peptides. The labeled samples are simultaneously analyzed by LC–MS/MS, and the quantification between samples is performed based on the relative intensities of the mass spectrometry peaks that correspond to differentially expressed proteins. This method yields a larger amount of data with higher quantitative accuracy compared to the data obtained via label-free quantification and can be used for comparing differential protein expression between samples from different interventions or time points [34]. DIA/SWATH is a data-independent acquisition (DIA) mode of scanning that differs from the conventional data-dependent acquisition (DDA) mode. In DIA/SWATH, secondary fragmentation of peptide precursors is performed, which allows for the relative/absolute quantification of proteins based on the resulting signals [35]. This approach provides richer and more accurate information compared to that provided by traditional DDA methods [31].

### Metabolomics

Metabolomics, a nascent discipline within the realm of omics, embodies the subsequent facet of systems biology. It is the omics method closest to the biological phenotype [36]. Based on the application of metabolomics, it can be divided into untargeted and targeted metabolomics [37]. The former methodology is centered on the comparative assessment of diverse small-molecule metabolites within samples, enabling the detection of distinct metabolites that vary across various groups. In contrast, the latter approach emphasizes the precise measurement of individual metabolites or a specific category, such as lipids, which can be used to validate differences in metabolic expression. Due to the complex physicochemical properties of metabolites, such as polarity and stability, the effectiveness and accuracy of metabolomics heavily rely on the selection of analytical platforms. The most popular analysis platforms for metabolomics include LC–MS, GC–MS, and nuclear magnetic resonance spectroscopy (NMR) [38]. Although NMR results are highly reproducible and the equipment is easy to operate, it can detect fewer metabolites than other analytical methods [38]. As no single separation method can cover the entire metabolome, LC–MS is commonly used for analyzing semi-polar metabolites, whereas GC–MS is preferred for analyzing volatile organic compounds and lipids [39]. Additionally, for untargeted metabolomics studies, multiple platforms are commonly used for concurrent detection to achieve a higher success rate by maximizing the coverage of metabolites [38].

### Microbiomics

Research on the gut microbiome has evolved significantly as a result of advancements in sequencing and mass spectrometry technologies, moving away from conventional culture methods to embrace the utilization of omics techniques [40]. Microbiomics is the study of all microorganisms in a specific environment. The 16S ribosomal RNA gene sequencing (16S rRNA-seq) and metagenomics techniques are most commonly used to study gut microbiota. The conventional approaches for 16S rRNA-seq and metagenomics rely on NGS. The 16S rRNA-seq technique targets specific hypervariable regions (e.g., V4 or V3–V4 regions) of the rDNA in microbial populations [41]. Metagenomics involves random fragmentation and PCR amplification of the total DNA extracted from microbial samples for sequencing [41]. As NGS-based 16S rRNA sequencing has short read lengths, it is unable to sequence the complete 16S rRNA gene. Hence, this technique can identify fewer bacteria at the species level than metagenomics [42]. However, these problems were resolved after the introduction of third-generation sequencing technologies, such as PacBio and Oxford Nanopore, which can be used to sequence the complete 16S rRNA gene [43]. Besides elucidating the composition of the gut microbiota, metagenomics can be employed to investigate microbial genes and their functional attributes through bioinformatics analysis. However, 16S rRNA sequencing and metagenomics cannot directly characterize the functional activities of microorganisms. Therefore, following the advancements in mass spectrometry technology, new areas of microbiome research, such as metaproteomics and metabolomics, have emerged [44, 45].

### Single-cell omics

Traditional omics technologies only provide information on dominant cell populations, often ignoring the information on cell populations with low abundance but high heterogeneity. Single-cell omics technologies can effectively address this issue by allowing the study of individual cells at a high resolution [46]. Single-cell omics methods have advanced considerably in recent years, and new technologies are being developed continuously. Among these methods, single-cell RNA sequencing (scRNA-seq) has received much attention. The key challenge in scRNA-seq lies in capturing individual cells, which was addressed with the development of droplet-based and microwell-based techniques and other technologies such as droplet-based single-cell RNA sequencing (Drop-seq), indexed droplet single-cell RNA sequencing (in Drop), 10 × Gem Code, and BD Rhapsody [47]. Single-cell cDNA amplification techniques, such as 3ʹ-end tagging, switching mechanism at the 5ʹ end of RNA template sequencing (Smart-seq), cellular indexing of transcriptomes and epitopes by sequencing (CEL-seq), etc., can also be used to amplify RNA from single cells [48]. With the advancement of single-cell omics, it is now possible to simultaneously detect multiple omics levels from individual cells. For example, techniques such as genome and transcriptome sequencing (G&T-seq) and TARGET-seq allow concurrent profiling of the whole transcriptome and genome of single cells [49, 50]. Techniques such as methylome and transcriptome sequencing from single-cells (scM&T-seq) and droplet-based single-nucleus chromatin accessibility and mRNA expression sequencing (SNARE-seq) have facilitated joint analysis of the transcriptome and the epigenome [51, 52]. Additionally, using the cellular indexing of transcriptomes and epitopes by sequencing (CITE-seq) and RNA end-associated purification sequencing (REAP-seq) techniques, researchers have combined single-cell transcriptomics with proteomics [53, 54].

## Application of omics in the study of TCM for antitumor purposes

### Transcriptomics

In transcription, genetic information flows from DNA to RNA. The study of the overall transcriptional landscape and changes in specific cell or tissue samples under certain conditions is known as transcriptomics. Transcriptomics can provide insights into the global transcriptional alterations in tumor cells or tissues before and after treatment with TCM. It can be used to understand the mechanisms underlying the antitumor effects of TCM (see Table 1 for details).

**Table 1:** Application of transcriptomics in studies on antitumor mechanisms of TCM

TCM type	Research object	Cancer type	Main technology	Main anti-tumor mechanism	References
Traditional Chinese medicine formulas	Xianlinglianxiafang	Breast cancer	RNA-seq	↓ The growth and metastasis of TNBC by ↑ the PPARγ/AMPK signaling pathway	[55]
	Xihuang pill	Lung cancer	RNA-seq	Through multiple pathways including tumor necrosis factor, estrogen, cGMP, etc	[56]
	Cinobufacini injection	Breast cancer	RNA-seq	Apoptosis and cell cycle arrest ↑ through the Pin1-TAZ pathway	[57]
	Biejiajian pill	Liver cancer	RNA-seq	↓ PDGFRβ signaling pathway, VEGF-A and HGF	[21]
	Yishen Qutong granule	Lung cancer	RNA-seq	↓ Oxidative stress-related protein HMOX1 expression	[58]
	Yangyinwenyang formula	Lung cancer	RNA-seq	↑ DC maturation and T cell proliferation and differentiation through the MAPK and NF-kB signaling pathways	[59]
Traditional Chinese herbs/monomers	*Trametes robiniophila* Murr	Breast cancer	cDNA microarray	↑ G0/G1 cell cycle arrest, ↓ RAD51,and interference with DNA repair to sensitization of radiotherapy	[60]
	*Aloe*	Colorectal cancer	RNA-seq	↑ Wnt/β-catenin pathway and ↓ Notch pathway	[61]
	*Coptidis rhizome*	Breast cancer	cDNA microarray	↑ IFN-b and exhibiting anti-tumor effects through autocrine pathways	[62]
	4-Methoxydalbergione (from *Dalbergia odorifera*)	Astroglioma	RNA-seq	↑ G2/M cell cycle arrest and cellular apoptosis through cell ↓ cycle-related genes (such as RRM2, BUB1, CDK1, etc.)	[63]
	Evodiamine (from *Evodia rutaecarpa*)	Esophageal squamous cell carcinoma	RNA-seq	↑ Mitotic cell cycle arrest through the CUL4A/p53/p21 axis and intrinsic-dependent apoptosis via Noxa	[64]
	Polydatin (from *Polygonum cuspidatum*)	Liver cancer	RNA-seq	↓ Expression of five genes involved in regulating the formation of the spindle midzone	[65]
	*N*-trans-Feruloyloctopamine (from garlic skin)	Liver cancer	RNA-Seq	↑ Apoptosis in liver cancer cells by modulating the proteins BBC3, DDIT3, CDKN1A, and NOXA	[66]
	Periplocin (from *Cortex periplocae*)	Pancreatic cancer	RNA-seq	↑ Autophagy by modulating the AMPK/mTOR pathway	[67]
	Cinobufagin (from *Bufonis Venenum*)	Human acute myeloid leukaemia	RNA-seq	↓ c-myc-related genes	[68]
	Neobractatin (from *Garcinia* *bracteata*)	Cervical cancer	RNA-seq	↑ G1/S phase and G2/M phase arrest through ↓ E2F1 and ↑ GADD45α	[69]
	Timosaponin AIII (from *Anemarrhenae Rhizoma*)	Tumor angiogenesis	RNA-seq	↓ Tumor angiogenesis through the VEGF/PI3K/Akt/MAPK signaling pathway	[70]
	Solamargine (from *Solanum nigrum L*.)	Multiple myeloma	RNA-seq	The anti-tumor effect is exerted by ↑ autophagy pathway	[71]
	Shikonin (from *Lithospermum erythrorhizon*)	Breast cancer	RNA-seq	↑ DUSP1, DUSP2 and exerting anti-breast cancer effect via the JNK and p38-MAPK pathways	[72]
	*N*-Butylidenephthalide (from *Radix Angelica Sinensis*)	Gastric cancer	RNA-seq	↑ REDD1, ↓ mTOR signaling pathway and intramitochondrial signaling pathways	[73]

“↑” represents increase, promote or up-regulate, while “↓” represents inhibit, suppress, decrease or down-regulate. The same goes for the tables in the back

#### Traditional Chinese medicine formulas

Chen et al. [21] performed RNA-seq to revealed that the Biejiajian pill might inhibit the development of liver cancer by downregulating the platelet-derived growth factor receptor-β (PDGFRβ) signaling pathway, as determined by RT-PCR and western blot analyses. The downstream proteins of the PDGFRβ signaling pathway, including phosphoinositide 3-kinase (PI3K), AKT, and GSK3β, were also detected, which indicated that they are involved in the mechanisms underlying the preventive effects of Biejiajian pill on the progression of liver cancer [21]. Zhao et al. [59] formulated the optimized prescription named Yangyinwenyang (YYWY) by refining kangfujin, demonstrating its favorable efficacy in combating lung cancer. The transcriptomics findings further revealed that the therapeutic properties of this compound are intricately linked to immune-mediated pathways, notably involving cytokine-cytokine receptor interaction as well as chemokine/NOD-like receptor/IL-17/NF-kB signaling pathways and finally proved the anti-tumor effect by promoting the proliferation and differentiation of mature DC-activated T cells [59].

#### Traditional Chinese herbs and monomers

Ding et al. [60] used the HTA 2.0 gene chip to investigate the radiosensitizing mechanism of Huai’er aqueous extract. The researchers found that Huai’er aqueous extract downregulated the expression of genes related to DNA repair, cell cycle, and cell division in MCF-7 cells. Further research found that the extract increased the sensitivity of breast cancer cells to radiation therapy by arresting the cell cycle and down-regulating radiation-sensitive mutant 51 (RAD51) [60]. One of the main chemical components of Jiangxiang is 4-methoxydalbergione (4-MDO). Transcriptome sequencing of U87 glioma cells treated with 4-MDO (0 µM or 5.0 µM for 12 h) showed that 158 genes were upregulated and 204 genes were downregulated [63]. Several genes associated with the cell cycle, including ribonucleotide reductase M2 (RRM2), minichromosome maintenance complex component 7 (MCM7), Budding Uninhibited by Benzimidazoles 1 Homolog Beta (BUB1B), Cyclin A2 (CCNA2), and CDK inhibitor 1 (CDK1), exhibited notable alterations. These findings suggested that 4MDO might be an effective therapeutic agent for treating glioma, considering and acts by blocking G2/M [63].

### Proteomics

Proteomics is used to investigate the entire protein composition and interactions within cells or tissues under specific conditions [74]. It not only provides qualitative and quantitative data on the overall protein content but also enables qualitative and quantitative analyses of post-translational modifications of proteins, including glycosylation, phosphorylation, ubiquitination, etc. [75]. Therefore, proteomics can be used as a complementary technology for studying the mechanisms of antitumor effects of TCM (details in Table 2).

**Table 2:** Application of proteomics in studies on antitumor mechanisms of TCM

TCM type	Research object	Cancer type	Main technology	Main anti-tumor mechanism	References
Traditional Chinese medicine formulas	Pien Tze Huang	Liver cancer	Protein chip	Modulating the secretion of inflammation-related cytokines, tumor growth pathways, and ↑ G2/M phase arrest	[76]
	Aidi injection optimal formula	Liver cancer/Colorectal cancer	TMT-based quantitative proteomics	Chen medicine can antagonize the activity of UPS lead by Jun medicine	[77]
Traditional Chinese herbs/monomers	*Camellia* *nitidissima* Chi	Colon Cancer	Label-free quantitative proteomics	Regulating iron death pathway-associated proteins such as GPX4 and HMOX1	[78]
	*Salvia chinensia* Benth	Esophageal cancer	TMT-based quantitative proteomics	↑ P-AMPK, P-ULK1, LC3 II/I and AMPK/ULK1 pathway	[79]
	*Antrodia Cinnamomea*	Liver cancer	2D-DIGE/MALDI-TOF MS proteomics	Regulating pathways related to protein folding, cellular cytoskeleton, and oxidation–reduction	[80]
	*Dendrobium nobile*	Lung cancer and liver cancer	SILAC quantitative proteomics	↑ ROS, ER stress and UPR and leading to autophagy and apoptosis	[81]
	*Lindera obtusiloba* Blume	Tumor angiogenesis	Protein chip	↑ Nibrin/NBS, ↓ Plk-1 and Cyclin E	[82]
	*Celastrus Orbiculatus* Vine	liver cancer	TMT / iTRAQ quantitative proteomics	↓ EphA2, tumor growth and angiogenesis	[83]
	Astragalus polysaccharide (from *Astragali Radix*)	Lung cancer	Label-free quantitative proteomics	Regulating the tumor microenvironment through the TLR4/MyD88/NF-κB signaling pathway	[84]
	Corilagin (from *Phyllanthus niruri* L)	Ovarian cancer	Protein chip and iTRAQ quantitative proteomics	↑ Apoptosis-related protein expression, ↓ CD44, STAT3 and glycolysis	[85]
	Triptolide (from *Tripterygium wilfordii*)	Lung cancer	iTRAQ quantitative proteomics	↑ MTA2 and EIF4A3, ↓ PHB, CDH1 and AIFM1, and regulating PARP1/AIF and Akt signaling pathways	[86]
	Celastrol (from *Tripterygium wilfordii*)	Cervical cancer	TMT-based quantitative proteomics	Confirming that GSTO1 and PDI are the targets	[87]
	Plectranthoic acid (from *Ficus microcarpa)*	Prostate cancer	Label-free quantitative proteomics	Through signaling pathways such as granulin A, endoplasmic reticulum stress, and mTOR	[88]
	Curcumin (from *Curcuma longa*)	Chronic myelogenous leukemia	SWATH quantitative proteomics	Through the miR-22/IPO7/HIF-1α axis	[89]
	SANT (monomer combination)	Breast cancer	Protein chip	↓ Blood vessel formation-related proteins (such as HB-EGF and IGFBP-9)	[90]

#### Traditional Chinese medicine formulas

Fan et al. [76] treated liver cancer xenograft Balb/c mice with administering Pien Tze Huang (0.234 g/kg) for 14 days and analyzed the phosphorylated proteins and signaling pathways in tumor tissues by conducting ingenuity pathway analysis (IPA). They identified 95 differentially phosphorylated proteins, which were enriched in multiple pathways related to inflammation, cancer growth, and cell cycle [76]. This study proved that Pien Tze Huang may exert its antitumor effects by regulating the secretion of inflammation-related cytokines and tumor growth pathways and inducing cell cycle arrest at the G2/M phase [76].

#### Traditional Chinese herbs and monomers

Chen et al. [78] used 4D-Label free proteomics analysis and found that 157 proteins were upregulated and 206 proteins were downregulated in HCT116 cells after treatment with Jinhua tea. The results of the gene ontology (GO) and kyoto encyclopedia of genes and genomes (KEGG) analyses indicated that these proteins were mainly associated with processes such as cell proliferation, apoptosis, and the cell cycle, and they were closely related to iron death pathways [78]. Li et al. [91] found that atractylenolide I (ATI) effectively inhibited the occurrence and development of colorectal tumors. Using proteomics techniques, they identified d-dopachrome tautomerase (D-DT) as a potential target for ATI in suppressing adenoma formation and confirmed that ATI can increase the expression of autophagy-related 7 (Atg7), Beclin, and microtubule-associated proteins 1A/1B light chain 3B (LC3B) in a dose-dependent manner. The findings showed that the inhibitory effects of ATI on the formation of colon cancer are associated with the activation of autophagy and the downregulation of D-DT [91].

### Metabolomics

Metabolomics can be used to characterize and quantify small-molecule metabolites in specific samples by combining separation techniques with high-resolution analysis techniques. Metabolomics has been extensively utilized to elucidate the mechanisms underlying the antitumor effects of TCM (details in Table 3).

**Table 3:** Application of metabolomics in studies on antitumor mechanisms of TCM

TCM type	Research object	Cancer type	Main technology	Main anti-tumor mechanism	References
Traditional Chinese hedicine formulas	Si Jun Zi Tang	Lung cancer	Q-TOF LC/MS	Regulating the lever of 2-oxoglutarate, tauroursodeoxycholic acid, oxidized glutathione, etc.	[92]
	Si-Ni-San	breast cancer	LC/MS	Inhibiting breast cancer growth by suppressing estradiol level via modulating FXR/EST signaling	[93]
	Shuihonghuazi formula	Liver cancer	HPLC/ESI-TOF–MS	Regulating the activity of PEMT and hemolytic phospholipase d, ↑ intake and utilization of linoleic acid and oleic acid and ↑ abnormal bile acid metabolism	[12]
	Xiaojin pills	Breast cancer	LC/MS	The ethanol extract, ↑ cholesterol sulfate, amino caproic acid glycocholic acid and ↓ l-glutamine	[94]
	Modified Si Jun Zi Tang	Gastric cancer	UHPLC-Q-TOF/MS	↓ LDH, GS, PCYT2 and regulating glycolysis, lipid metabolism, etc	[95]
Traditional Chinese herbs/monomers	*Glycyrrhiza glabra*	Nasopharyngeal carcinoma	GC/MS	↓ Amino acids and lipid metabolites and enriching in multiple amino acid and lipid metabolic pathways	[96]
	Frankincense -myrrh	Multiple myeloma	UPLC/Q-TOF–MS	Regulating the JAK/STAT signaling pathway and suppressing cellular metabolism	[97]
	*Spica Schizonepetae*	Lung cancer	UPLC-QTOF-MS	Regulating metabolism of glycerophospholipids, purines, histidine, and arachidonic acid, etc	[98]
	BFI, CFI (from Chansu)	Liver cancer	UHPLC-MS/MS	Exerting synergistic effects through the metabolic pathways of phenylalanine, α-linolenic acid, and amino acids	[99]
	BFI, CFI (from Chansu)	Liver cancer	LC − MS/MS	Exerting synergistic effects through methionine metabolism	[100]
	Magnoline (from *Phellodendri amurensis cortex*)	Prostate cancer	UHPLC-HDMS/MS	The significant impact of 12 metabolites (such as phenylalanine, tyrosine, etc.)	[101]

#### Traditional Chinese medicine formulas

Zhang et al. [93] conducted a study which demonstrated the ability of Si-Ni-San to inhibit the growth and metastasis of breast cancer by activating estrogen sulfotransferase (EST) to promote the sulfation of estradiol. Furthermore, the study identified changes in 78 metabolites, including taurochenodeoxycholic acid and taurocholic acid, in the livers of mice before and after drug administration through metabolomics, with a primary focus on the bile acid metabolism pathway [93]. The research has conclusively demonstrated that Si-Ni-San can activate the expression of farnesoid X receptor (FXR) through bile acids, thereby inhibiting the transcription of estrogen sulfotransferase (EST) mediated by hepatocyte nuclear factor 4a (HNF4a) in liver cells, ultimately resulting in elevated estradiol levels [93]. Liu et al. [94] found that the ethanol extract of the xiaojin pill has stronger anti-breast cancer effects than the water extract on a BALB/c nude mouse model of breast cancer metastasis and on cells in vitro. The results of metabolomics analysis showed that cholesterol sulfate and 6-aminohexanoic acid levels were significantly higher in the ethanol extract group, whereas l-glutamine and glycocholic acid levels were significantly lower and the differential metabolites were enriched in pathways related to glycerophospholipid metabolism, alanine metabolism, and aspartate metabolism [94]. The findings of that study provided a theoretical basis for changing the administration route of xiaojin Pill from “water-based” to “alcohol-based”.

#### Traditional Chinese herbs and monomers

Wang et al. [98] demonstrated the anti-tumor effects of *Spica Schizonepeta* on Lewis lung carcinoma mice. Using QTOF-MS/MS-based metabolomics technology, they showed that treatment with *Spica Schizonepetae* extract resulted in changes in the levels of 23 endogenous metabolites (including uridine-3-ribonucleotide, indole-3-acetic acid, adenine, etc.) in A549 cells, which were enriched in 16 pathways, including glycerophospholipid metabolism, purine metabolism, histidine metabolism, etc. [98]. Zhang et al. [100] demonstrated that the combined use of bufalin (BFL) and cinobufagin (CBF) has a stronger anti-cancer effect in vitro. Then LC–MS metabolomics technology was used to confirm that the synergistic enhancement mechanism of BFL and CBF was associated with the methionine metabolism pathway, revealing the potential of BFL and CBF combination for clinical treatment of liver cancer.

### Microbiomics

The gut microbiota, consisting of beneficial and pathogenic bacteria, represents the largest microbial community within the human body. Several studies have shown that the dysregulation of the gut microbiota composition and function is closely associated with the occurrence and development of cancer [102]. TCM can exert indirect anti-tumor effects by improving the gut microbiota, which are usually accompanied by changes in metabolic functions and cancer immune activation. Therefore, when elucidating the mechanism underlying the antitumor activity of TCM through gut microbiota, microbiomics and metabolomics are frequently employed in conjunction with each other. The combined application of these two omics is shown in Table 4.

**Table 4:** Application of microbiomics in studies on antitumor mechanisms of TCM

TCM type	Research object	Cancer type	Main technology	Main anti-tumor mechanism	References
Traditional Chinese medicine formulas	Sini decoction	Colorectal cancer	16S rRNA-seq	Altering the composition of gut microbiota and modulating intestinal immunity	[103]
	Yi-Yi-Fu-Zi-Bai-Jiang-San	Colorectal cancer	16S rRNA-seq	Reshaping the gut microbiota, such as Bacteroides fragilis and Lachnospiraceae, while suppressing Treg cells	[104]
	Wenzi Jiedu recipe	Colorectal cancer	16S rRNA-seq	↑ Osci llibacter and Bacteroides_acidifacien, and CD8+ T cells	[105]
	Jiawei Xiaoyao san	Liver cancer	16S rRNA-seq and Metabolomics	↑ Beneficial bacteria, ↓ harmful bacteria, regulating primary bile acid biosynthesis and phenylalanine metabolism	[106]
	Gegen Qinlian decoction	Colorectal cancer	16S rRNA-seq and Metabolomics	↑ s__Bacteroides_*acidifaciens*, regulating glycerophospholipid and sphingolipid metabolism and activating the immune system	[107]
Traditional Chinese herbs/monomers	Curcumin (from *Curcuma Longa*)	Colorectal cancer	16S rRNA-seq	↓ Bacteroidaceae, Ruminococcaceae, Firmicutes, and ↑ Lactobacillaceae Bifidobacteriaceae	[108]
	glycyrrhiza polysaccharide (from *Glycyrrhiza uralensis* Fisch.)	Colorectal cancer	16S rRNA-seq	GCP can significantly alter the gut microbiota to exert anti-tumor effects, which was further validated by FMT experiments	[109]
	*Panax Ginseng*	Liver cancer	16S rRNA-seq and Metabolomics	Modulating gut microbiota composition and its close association with bile acids, unsaturated fatty acids	[110]
	*Astragalus Mongholicus* Bunge-*Curcuma Aromatica* Salisb	Colorectal cancer	16S rRNA-seq and Metabolomics	Enhancing gut microbiota diversity and increasing the levels of short-chain fatty acids such as propionic acid and butyric acid	[111]
	*Tetrastigma hemsleyanum* Diels et Gilg	Colorectal cancer	16S rRNA-seq and Metabolomics	↑ Beneficial bacteria, ↓harmful bacteria, and restoring abnormal metabolites in the feces	[112]
	Ginsenoside Rg3 (from *Panax Ginseng*)	Liver cancer	16S rRNA-seq and Metabolomics	↑ Bacteroidetes and Verrucomicrobia, ↓ Firmicutes and Measuring indole-3-propionic acid and urea	[113]
	*Ginseng* polysaccharides (from *Panax Ginseng*)	Lung cancer	16S rRNA-seq and Metabolomics	↑ Parabacteroides distasonis and Bacteroides vulgatus, ↑ butyric acid, ↓ l-tryptophan and Kyn/Trp to enhance the effect of αPD-1	[114]

#### Traditional Chinese medicine formulas

Lv et al. [107] conducted in vivo experiments and found that the anti-PD-1 immune therapeutic efficacy was significantly enhanced by Ge-Gen-Qin-Lian decoction (300 mg/kg). Through 16S rRNA sequencing, Akkermansia (s__*Bacteroides*_*acidifaciens* and s__*uncultured*_*organism*_g__*norank*_f__*Bacteroidales*_S24-7_*group*) were significantly enriched in the combination treatment group, whereas plasma metabolomics analysis showed substantial changes in sphingolipid and glycerophospholipid metabolism [107]. The combined treatment group also exhibited higher levels of interferon gamma (IFN-γ) and interleukin-2 (IL-2), lower levels of programmed death 1 (PD-1), and greater infiltration of CD8+ T cells in the tumor tissues of mice [107]. Liver stagnation and spleen deficiency are the main syndromes of TCM in the diagnosis of liver cancer. Li et al. used 16S rRNA sequencing and plasma metabolomics to investigate the impact of modified Iiawei xiaoyaowan (JWXY) on the gut microbiota composition in a rat model of liver cancer with liver depression and spleen deficiency syndrome [106]. The intervention by JWXY resulted in an increase in the abundance of Firmicutes and a decrease in the abundance of Bacteroidetes [106]. They conducted a metabolic pathway analysis and found changes in 11 pathways, including primary bile acid biosynthesis and phenylalanine metabolism, compared to the state of those pathways in the model group [106].

#### Traditional Chinese herbs and monomers

*Astragalus mongholicus* Bunge-*Curcuma aromatica* Salisb extract (ACE) is a commonly used drug in the clinical treatment of tumors. Gu et al. conducted 16S rRNA sequencing and targeted metabolomics analysis on fecal samples from colon cancer-bearing mice treated with ACE extract and found that ACE effectively inhibited the growth of harmful bacteria like *Streptococcus* and *Enterococcus*, while enhancing the presence of beneficial bacteria such as *Lactobacillus* and *Prevotella* [111]. ACE increased the levels of short-chain fatty acids such as propionic acid and butyric acid, and the anti-colorectal cancer effect of ACE was ultimately attributed to the inhibition of the stromal cell-derived factor-1/C-X-C motif chemokine receptor 4 (SDF-1/CXCR4) signaling pathway [111]. *Ginseng* polysaccharides (GPs) exert antitumor activity by modulating the immune system. Huang et al. [114] conducted a fecal microbiota transplantation (FMT) experiment and found that the combination treatment of GPs with an anti-programmed death-1 antibody (αPD-1) monoclonal antibodies (mAbs) led to the reshaping of the intestinal microbiota in PD-1 non-responsive mice by upregulating the levels of *Bacteroides dorei* and *Bacteroides vulgatus*. In addition, GPs increased the level of butyric acid and decreased the l-kynurenine/tryptophan (Kyn/Trp) ratio [114]. These changes converted mice that were non-responsive to immunotherapy into responsive ones, thus enhancing the antitumor response to αPD-1 monoclonal antibodies [114].

### Single-cell omics

Single-cell omics diverge from conventional omics as they enable the precise acquisition of molecular information, such as information on genes, mRNA, and proteins, at the level of individual cells. This approach can be used to study tumor cell heterogeneity [115], the tumor microenvironment (TME) [116], tumor resistance [117], and other related areas. Due to the significant progress in single-cell RNA sequencing (scRNA-seq) technology, it has become an important methodology in elucidating the complex antitumor molecular mechanisms of TCM. Wu et al. used a combined strategy of traditional RNA-seq and scRNA-seq to examine the impact of Compound Kushen Injection (CKI) in conjunction with paclitaxel (PTX) on the tumor microenvironment (TME) of triple-negative breast cancer (TNBC) [118]. By sequencing 174,434 cells, the researchers found that the combined treatment of CKI and paclitaxel PTX significantly enhanced immune responses and facilitated T-cell activation, resulting in a higher proportion of cytotoxic T cells and NK cells in tumor tissue [118]. The discovery underscored the ability of CKI to boost the efficacy of chemotherapy by activating immune responses [118]. WZ35, a derivative of curcumin, can rapidly induce ROS production and inhibit the metastasis of gastric cancer (GC) cells. Chen et al. [119] employed a metabolomics methodology to demonstrated that WZ35 can regulate amino acid metabolism and deplete GSH through multiple pathways. To further investigate the mechanism, the researchers analyzed single-cell sequencing data from 33,031 cells of 128 gastric cancer patients. They found a strong correlation between the expression of yes-associated protein (YAP) and AXL receptor tyrosine kinase (AXL) genes and EMT, as well as, amino acid metabolism [119]. Further experiments showed that WZ35 effectively downregulated the levels of AXL and YAP proteins and upregulated the expression of the metastasis-associated protein N-cadherin [119]. Compound Andrographis Indica Tablet is an oral arsenic agent widely used in the treatment of acute promyelocytic leukemia (APL). To explore the scientific knowledge of As_2_O_3_-indigo formula compatibility, Zhang et al. chose arsenic trioxide (As_2_O_3_: A), tanshinone IIA (T) and indirubin (I) as representative active compounds ofrealgar, indigo naturalis, and *Salvia miltiorrhiza* and treated leukemic mice with A, AT, AI, and ATI, followed by single-cell transcript sequencing analysis of bone and bone marrow stromal cells from each group [120]. The combination of ATI was found to have the strongest therapeutic effect in the mouse APL model and to be most sensitive to Lepr-MSCs, OLC, and BMEC cell populations compared to the A, AT, or AI groups [120]. Based on the scRNA-seq data, ATI can regulate the gene expression related to osteogenic differentiation, adipogenic differentiation, and endothelial cell migration of bone marrow mesenchymal stromal cells, thereby improving the expression of normal hematopoiesis-related genes and adverse prognosis in leukemia mice Lepr-MSCs, OLCs, and BMECs [120].

## Application of multi-omics in studies of TCM for antitumor purposes

With the continuous advancements in single-omics technologies, the amount of data obtained has increased significantly. However, single-omics approaches are unable to fully characterize biological features as biological processes are complex and integrated. Therefore, further integration and analysis of multi-omics data, with mutual validation at multiple molecular levels, can more systematically elucidate the overall regulatory mechanisms of TCM in antitumor effects (details in Table 5).

**Table 5:** Application of multi-omics in studies on antitumor mechanisms of TCM

TCM type	Research object	Cancer type	Omics type	Main anti-tumor mechanism	References
Traditional Chinese medicine formulas	Weining granule	Gastric cancer	Epigenomics, transcriptomics, and proteomics	Relevant targets: SOD2, MMP1, SRXN1, NOTCH1, MAPK14 Relevant pathways; PI3K-Akt, MAPK	[121]
	Yiqi Sanjie formula	Colorectal cancer	Proteomics and metabolomics	Changing 57 proteins and 37 metabolites which are enriched in inflammation, sphingolipid metabolism, and cholesterol metabolism-related pathways	[122]
	Xian-Lian-Jie-Du decoction	Colorectal cancer	Transcriptomics and proteomics	↑ Mfsd2a, Ccdc85c and ↓B cells in the tumor microenvironment	[123]
	Canmei formula	Colorectal cancer	Transcriptomicsand proteomics	Regulating LHPP through the PI3K/Akt signaling pathway	[124]
Traditional Chinese herbs/monomers	*Marsdenia tenacissima* (Roxb.) Moon	Liver cancer	Transcriptomics and metabolomics	Targeting P53, JAK-1, and HIF1α to exert anti-hepatocellular carcinoma activity	[125]
	*Inonotus hispidus*	Liver cancer	Transcriptomics, and proteomics	Five key genes: Lilrb4a, Nrp1, Gzma, Gstt1, and Pdk4	[126]
	Shikonin (from *Lithospermum erythrorhizon*)	Hematological malignancies	Transcriptomics, and proteomics	↓ IGF1R-Akt-mTOR pathway by ↓ AKT phosphorylation and IGF1R kinase activity	[127]
	Cepharanthine (from *Stephania cepharantha* Hayata)	Liver cancer	Transcriptomics and metabolomics	Altering 168 genes which enriched in metabolic pathways (such as multiple amino acid levels)	[128]
	Cinobufagin (from Chansu)	Liver cancer	Transcriptomics and metabolomics	Interfering with metabolic reprogramming, including lipid, amino acid, carbohydrate, and nucleotide metabolism	[129]
	Bufalin (from Chansu)	Liver cancer	Transcriptomics and metabolomics	Regulating the ATP1A1/CA1 axis and downregulating the SREBP-2/FASN/ACLY pathway	[130]

### Traditional Chinese medicine formulas

Wening granules (WNG) is a well-known formulation that is clinically used for treating gastric cancer. Liang et al. [121] integrated reduced representation bisulfite sequencing (RRBS), TMT-based quantitative proteomics, and RNA-seq to construct a comprehensive molecular landscape of SGC-7901 cells treated with WNG. They identified 1249 differentially expressed genes (DEGs), 191 differentially abundant proteins (DAPs), and 8293 differentially methylated regions [121]. Through comprehensive analysis of the RNA and protein data, 95 genes were identified to exhibit significant changes at both levels, and further protein–protein interaction (PPI) network analysis confirmed the top eight candidate genes, including recombinant human superoxide dismutase (SOD2), heme oxygenase 1 (HMOX1), and matrix metalloproteinase-1 (MMP1) [121]. Similarly, by integrating RNA and DNA-related data, eight hub genes were selected, such as RNA polymerase II subunit F (POLR2F), peptidylprolyl isomerase like 4 (PPIL4), and retinoblastoma-binding protein 7 (RBBP7) [121]. By integrating the final data, SOD2, sulfiredoxin 1 (SRXN1), neurogenic locus notch homolog protein 1 (NOTCH1), and mitogen-activated protein kinase 14 (MAPK14) were predicted to be the target genes for the anti-gastric cancer effects of WNG [121].

### Traditional Chinese herbs and monomers

Li et al. [125] studied *Marsdenia tenacissima* extracts (MTE) using LC–MS and identified three compounds (TI, TG, and TH) that contributed the most to the circulating components of mouse serum after they were administered. Then, researchers identified differential genes and metabolites by transcriptomics and metabolomics, respectively [125]. The five common targets, including tyrosine-protein kinase (JAK-1), PI3K, and hypoxia-inducible factor (HIF-1), were selected by combining the predictions of targets through bloodstream components, omics-related targets, and liver cancer-related targets [125]. In addition, molecular docking analysis showed that TI, TG, and TH could target p53, HIF1α, and JAK1, respectively, and collectively exert anti-hepatocellular carcinoma effects [125]. These findings highlighted the characteristics of TCM, where multiple components act on multiple targets synergistically. Fan Feng et al. [128] conducted in vivo and in vitro experiments to demonstrate that Triptolide has significant anti-hepatocellular carcinoma effects. Then, the results of the RNA-seq analysis showed that treatment with Triptolide affected the expression of 166 genes (53 upregulated and 113 downregulated) in HCCLM3 cells; these genes enriched pathways related to necrosis and metabolism and metabolomics analysis showed that metabolites such as 4-aminobutyric acid and l-5-oxoproline were significantly reduced [128]. These results suggested that Triptolide exerts antitumor effects by modulating various amino acid metabolic pathways [128].

## Application of omics-network pharmacology in studies of TCM for antitumor purposes

Network pharmacology (NP) is a scientific discipline based on systems biology that integrates biological networks to reveal the interactions among drugs, genes, targets, and diseases [131]. The intricate composition and multi-target nature of TCM align with the comprehensive, systematic, and integrative features of NP. Therefore, NP is an important tool for investigating the mechanism of action of TCM [132]. However, there are concerns about the reliability of acquiring data on the components of traditional Chinese herbs and their target genes from databases using traditional network pharmacology. The integration of omics and NP not only increases the authenticity of the NP data but also compensates for the limitations in the correlation between herbal components and target genes in omics studies. Hence, this strategy is extensively used, as shown in Table 6.

**Table 6:** Application of omics-network pharmacology in studies on antitumor mechanisms of TCM

TCM type	Research object	Cancer type	Omics type	Main anti-tumour mechanism	References
Traditional Chinese medicine formulas	BL 02	Lung cancer	NP and transcriptomics	The anti-tumor activity may be directly attributed to the interaction of mangiferin and 5-methylcoumarin-4-cellobioside with Rap1	[133]
	Chang qing formula	Colorectal cancer	NP and transcriptomics	↓ IL-17a, MMP9, and NF-κB/IL-6/STAT3 signaling pathway	[134]
	Xihuang pill	Breast cancer	NP and proteomics	Modulating the PI3K-AKT signaling pathway	[135]
	Weijing decoction	Lung cancer	NP and metabolomics	Active ingredient: quercetin ↓ PRKCA and sphingolipid signaling pathways	[136]
	Xiaojijinzhan decoction	Colorectal cancer	NP and transcriptomics	Inhibiting the metastatic and invasive abilities of colorectal cancer by modulating the VDR-TGF-β signaling pathway	[137]
	Mufangji decoction	Lung cancer	NP and transcriptomics	Active ingredient: sinomenine and dehydrocostus lactone ↑ MPO and immune pathways	[138]
	Bushen-Jianpi-Jiedu decoction	Colorectal cancer	NP and transcriptomics and proteomics	Combined with oxaliplatin can regulate various plasma proteins (↑ ZEB2 and CAT; ↓ IL-1A and CD5L)	[139]
	Feiyanning formula	Lung cancer	NP and metabolomics	Promoting metabolism and activating mitochondrial pathways to induce apoptosis in cancer cells	[140]
	Qingyihuaji formula	Pancreatic cancer	NP and proteomics	Active ingredient: quercetin ↓ MAPK/ERK and PI3K/Akt/mTOR signaling pathways	[141]
	Gegen Qinlian fecoction	Colorectal cancer	NP and transcriptomics	The active ingredients in Jun and Chen medicine exhibit a synergistic effect on the Wnt signaling pathway	[142]
	Xihuang pill	Lung cancer	NP and transcriptomics	Targeting CACNA1C to modulate the TME	[143]
Traditional Chinese herbs	*Astragalus membranaceus*	Liver cancer	NP and transcriptomics	Active ingredient: quercetin ↓ MT1G to activate iron-dependent cell death	[144]
	*Rhus chinensis* Mill	Colorectal cancer	NP and metabolomics	Targeting ENO1 and ALDOA, and regulating glycolysis and glutamine metabolism pathways	[145]
	*Codonopsis* *pilosula*	Liver cancer	NP and transcriptomics	Active ingredient: mogroside, capsaicin, and sulforaphane ↑ HMOX1 and modulating of mineral uptake pathways	[146]
	*Radix* *Ophiopogonis*	Nasopharyngeal carcinoma	NP and proteomics	Targeting VEGFA, TP53, and HSPA8 and modulating PI3K-Akt, Wnt, cAMP signaling pathways	[147]
	*Taraxacum mongolicum*	Breast cancer	NP and metabolomics	Regulating the cell cycle and metabolic pathways	[148]

### Traditional Chinese medicine formulas

Li et al. [136] conducted in vivo experiments and found that WJT (an herbal formula) inhibited the development of Lewis lung carcinoma and considerably prolonged the survival of mice. Then, researchers utilized NP to determine quercetin as the principal active constituent of WJT, with protein kinase C alpha (PRKCA) being its primary target [136]. Meanwhile, metabolomics analysis indicated that WJT partially rectified the metabolic imbalance induced by lung cancer; in the analysis, sphingosine-1-phosphate was identified as a prominent differential metabolite [136]. These results indicated that WJT exerts its antitumor effects primarily by inhibiting the PRKCA/SPHK/S1P signaling pathway and the anti-apoptotic signaling pathway [136]. By implementing NP, the Feiyanning formula (FYN) was found to exert anti-lung cancer effects by activating the mitochondrial pathway [140]. Meanwhile, the results of metabolomics showed that FYN reduced the levels of putrescine and agmatine and increase in ATP utilization in A549 cells [140]. Following FYN treatment in vitro, the mitochondrial membrane potential decreased and the expression of calcium-binding proteins increased on the cell surface, which further supported the predicted results [140].

### Traditional Chinese herbs

Qu et al. [148] identified 12 bioactive constituents in Dandelion using LC-Q-TOF/MS and found 50 targets associated with TNBC that were significantly enriched in cell cycle and metabolism-related pathways through NP analysis [148]. The results of a metabolomics analysis revealed 22 altered metabolites, including changes in pathways related to arginine and proline metabolism, which matched the predicted pathways from the network pharmacology analysis [148]. These findings elucidated the anti-breast cancer effects of Dandelion through the modulation of cell cycle and metabolism pathways.

## Discussion and prospects

The progression from normal cells to tumor cells is an extremely complex process, during which tumor cells acquire various capabilities, including but not limited to sustained proliferation, resistance to programmed cell death, invasion and metastasis abilities, cellular metabolic reprogramming, and evasion of the immune system through continuous mutations [1]. The development of tumors is also closely associated with changes in the tumor microenvironment and the gut microbiota [149, 150]. Unlike Western medical strategies for tumor treatment, traditional Chinese herbs and herbal formulations have highly complex chemical compositions, which often act on multiple targets and inhibit tumor development through multiple pathways. For example, ginseng contains multiple antitumor active components, such as ginsenosides (Rh2, Rg3, Rg5, etc.), ginseng polysaccharides, and ginsenoids. It exerts antitumor effects or enhances the antitumor effect of chemotherapeutic agents through various mechanisms, including the inhibition of proliferation, invasion, and metastasis, improvement of the TME, immune modulation, and regulation of the gut microbiota [114, 151, 152]. Therefore, when elucidating the mechanisms underlying the antitumor effects of TCM, especially complex formulas, a research approach based on a single target pathway or characterization of the activity of a single active component cannot represent their complex network of antitumor mechanisms.

Due to continuous advancements in separation and analysis technologies, omics techniques have provided new ways to study the antitumor mechanisms of TCM. High-throughput transcriptomics and proteomics can aid researchers in identifying key anti-cancer pathways or candidate targets in TCM treatments for cancer. For instance, Chen et al. [21] identified PDGFRβ as the target of Biejiajian pill in liver cancer using RNA-seq technology. Li et al. [91] revealed through proteomics that the anti-colorectal cancer effects of atractylenolide I (ATI) are closely related to autophagy activation and downregulation of d-DT. Microbiomics such as 16S rRNA-seq and metagenomics can provide information on the different bacterial genera and species present in the gut microbiota, including the relative abundance of beneficial, harmful, and other microorganisms. This information is valuable for assessing the impact of TCM interventions on the gut microbiota, which has been shown to be a crucial pathway for its anti-tumor effects [41]. For example, Zhang et al. [109] demonstrated that Glycyrrhiza uralensis polysaccharide (GCP) exerts its anti-tumor effects by closely interacting with the gut microbiota through 16S rRNA-seq and FMT experiments. Additionally, metabolomics elucidates the mechanisms of TCM's anti-tumor effects by analyzing changes in metabolites from tumor cells/tissues or the gut microbiota. This is of significant importance for studying how TCM exerts its anti-tumor effects through various metabolic pathways, particularly lipid metabolism, or through microbiota-derived metabolites. Single-cell omics focuses on the heterogeneity among individual cells within a sample, enabling the identification of different cell subpopulations and their unique functions. This is of profound significance for both tumor and immune cells. Currently, many researchers are beginning to explore the role of single-cell omics in the study of TCM's anti-tumor and immune effects [118–120]. In summary, omics technologies have greatly advanced the field of TCM for cancer treatment, providing new methods and perspectives.

Single-omics technologies have certain limitations, as biological systems are interconnected and dynamic. Interpreting the anti-tumor effects of TCM solely at the level of transcription or protein often overlooks the regulatory relationships between these layers. Multi-omics technologies can reveal the intricate therapeutic mechanisms of traditional Chinese medicine for various diseases from different levels and illustrate the interactions among them [153]. In cancer research, the combination of transcriptomics and proteomics is common. Integrating DEGs and DAPs (usually in objects with significant changes at both levels) and further analysis (such as PPI) are used to explore potential traditional TCM anti-tumor candidate targets for subsequent research. A study by Liang et al. predicted multiple anti-tumor targets of Wenjing granules (such as SOD2, NOTCH1) through the combined analysis of the two omics [121]. The protein and metabolite networks are closely interrelated. In tumors, mutations in metabolic enzymes can lead to metabolic abnormalities, while metabolites can also affect protein expression or activity. For example, mutations in phosphofructokinase-1 (PFK-1) can lead to the well-known ‘Warburg effect’ [154]. Additionally, abnormal expression of fatty acid synthase (FASN) and cholesterol ester transfer protein (CETP) can result in disrupted lipid metabolism in tumors, thereby promoting survival [155, 156]. Therefore, the integration of transcriptomics/proteomics and metabolomics is commonly employed to investigate the intricate regulation between the proteome and metabolome in tumors following TCM interventions [122, 125]. Moreover, the combined use of microbiomics and metabolomics has successfully deciphered the changes in gut microbiota metabolism following TCM intervention (particularly bile acid and fatty acid metabolism), further elucidating the importance of gut microbiota in the anti-tumor effects of TCM. In conclusion, multi-omics techniques are of great significance in the study of the anti-tumor effects of TCM, and will effectively elucidate the complex mechanisms involved in the anti-tumor actions of TCM.

Although omics technologies are powerful tools for determining the mechanisms underlying the antitumor effects of TCM, they have some limitations. Firstly, although many researchers have determined the antitumor effects of TCM and the underlying mechanisms through multi-omics approaches, most studies have focused on independent analysis of each omics level and thus deeper insights into the regulatory relationships between different omics layers are needed. Secondly, although omics technologies can be used to identify potential antitumor molecular targets of TCM, there is limited research exploring which active ingredients may bind to them. Lastly, cutting-edge omics technologies such as single-cell/spatial omics and SMRT-seq have not yet been integrated with studies on the antitumor effects of TCM.

To summarize, omics technologies serve as powerful tools for elucidating the mechanism of action of TCM, and their application has already shown promising results. Meanwhile, the field of omics technologies are currently experiencing rapid advancement, with the emergence of innovative techniques such as single-cell omics and spatial omics. In the foreseeable future, these advancements are anticipated to offer substantial assistance in elucidating the anti-tumor mechanisms of Chinese medicine.

## Data Availability

All data used in this systematic review are fully available in the public domain.

## Abbreviations

ACE: *Astragalus mongholicus* Bunge-*Curcuma aromatica* Salisb extract
Atg7: Autophagy-related 7
ATI: Atractylenolide I
BFL: Bufalin
BUB1B: Budding uninhibited by benzimidazoles 1 homolog beta
CBF: Cinobufagin
CDK1: CDK inhibitor 1
cDNA: Complementary deoxyribonucleic acid
CEL-seq: Cellular indexing of transcriptomes and epitopes by sequencing
cGMP: Cyclic guanosine monophosphate
CITE-seq: Cellular indexing of transcriptomes and epitopes by sequencing
CKI: Compound kushen injection
CXCR4: C-X-C motif chemokine receptor 4
DAPs: Differentially abundant proteins
DDA: Data-dependent acquisition
d-DT: D-Dopachrome tautomerase
DEGs: Differentially expressed genes
DIA/SWATH: Data independent acquisition/sequential window acquisition of all theoretical spectra
DNA: Deoxyribonucleic acid
Drop-seq: Droplet-based single-cell RNA sequencing
FYN: Feiyanning formula
G&T-seq: Genome and transcriptome sequencing
GC–MS: Gas chromatography-mass spectrometry
GO: Gene ontology
GPs: Ginseng polysaccharides
GS: Glutamine synthetase
HIF-1: Hypoxia-inducible factor 1
HMOX1: Heme oxygenase 1
IFN-γ: Interferon gamma
IL-2: Interleukin-2
In Drop: Indexed droplet single-cell RNA sequencing
IPA: Ingenuity pathway analysis
JAK-1: Tyrosine-protein kinas
KEGG: Kyoto encyclopedia of genes and genomes
Kyn/Trp: L-Kynurenine/tryptophan
LC3B: Microtubule-associated proteins 1A/1B light chain 3B
LC–MS: Liquid chromatography-mass spectrometry
LDH: Lactate dehydrogenase
MAPK: Mitogen-activated protein kinase
MAPK14: Mitogen-activated protein kinase 14
MCM7: Minichromosome maintenance complex component 7
MMP1: Matrix metalloproteinase-1
MTE: *Marsdenia tenacissima* Extracts
NF-κB: Nuclear factor kappa-B
NMR: Nuclear magnetic resonance spectroscopy
NGS: Next generation sequencing
NOTCH1: Neurogenic locus notch homolog protein 1
NP: Network pharmacology
PCR: Polymerase chain reaction
PD-1: Programmed death 1
PDGFRβ: Platelet-derived growth factor receptor-β
PI3K: Phosphoinositide 3-kinase
POLR2F: RNA polymerase II subunit F
PPIL4: Peptidylprolyl isomerase like 4
PRKCA: Protein kinase C alpha
PTX: Paclitaxel
RAD51: Radiation-sensitive mutant 51
RBBP7: Retinoblastoma-binding protein 7
REAP-seq: RNA end-associated purification sequencing
RRBS: Reduced representation bisulfite sequencing
RRM2: Ribonucleotide reductase M2
scRNA-seq: Single-cell RNA sequencing
scM&T-seq: Methylome and transcriptome sequencing from single-cells
SDF-1: Stromal cell-derived factor-1
SILAC: Stable-isotope labelling by amino acids in cell culture
Smart-seq: Switching mechanism at the 5ʹend of RNA template sequencing
SOD2: Recombinant human superoxide dismutase
SRXN1: Sulfiredoxin 1
TCM: Traditional Chinese medicine
TME: Tumor microenvironment
TMT/iTRAQ: Tandem mass tags/isobaric tag for relative absolute quantitation
TNBC: Triple-negative breast cancer
WNG: Wening granules
XHP: Xihuang pill
YAP: Yes-associated protein
αPD-1: Anti-programmed death-1 antibody
16S rRNA-seq: 16S ribosomal RNA gene sequencing
4-MDO: 4-Methoxydalbergione
